# A Treatment Algorithm for Free Vascularized Bone Reconstruction in Rare Large Osseous Defects Involving the Wrist

**DOI:** 10.3390/life14091099

**Published:** 2024-08-31

**Authors:** Johannes Tobias Thiel, Maximilian Bamberg, Adrien Daigeler, Johann Fontana, Sebastian Hoffmann, Claudius Illg, Jonas Kolbenschlag, Dominik Steiner, Henrik Lauer

**Affiliations:** 1 Department of Hand, Plastic, Reconstructive and Burn Surgery, BG Trauma Center Tübingen, University of Tübingen, 72076 Tübingen, Germany; 2 Department of Anesthesiology and Intensive Care Medicine, BG Trauma Center Tübingen, Schnarrenbergstr. 95, 72076 Tübingen, Germanyjfontana@bgu-tuebingen.de (J.F.)

**Keywords:** bone defects, upper extremity, microsurgery, vascularized bone transplants

## Abstract

Large bone defects of the distal radius and/or carpus following tumor resection, trauma, or infection are extremely rare. There are few case reports and series in the literature on the reconstruction approaches required in such cases. Therefore, large studies cannot be used to guide the therapeutic decisions of reconstructive plastic and hand surgeons. The objective of this study is to propose a treatment algorithm to predict the functional outcome and quality of life for the different techniques of free vascularized bone reconstruction in the interval between the distal radius, the carpus, and/or the proximal metacarpal. The algorithm was developed based on our own case studies and the few treatment approaches described in the literature. It can be applied to rare cases of massive bone defects in the wrist. The flowchart enables surgeons to develop an individualized reconstruction concept for various intervals of bone defects in the area of the distal radius and proximal metacarpal bones. Ultimately, the treatment algorithm aims to maximize future quality of life (QoL) and function of the distal upper extremity in rare cases of massive wrist-bone defects.

## 1. Introduction

The occurrence of large osseous defects of the distal radius and/or carpus is exceedingly rare. The primary causes of such defects are tumors and deep infections [1,2,3,4]. For instance, the literature contains descriptions of various conditions, including giant cell tumors, osteomyelitis, and other less-common causes such as failed wrist prosthesis or trauma [5,6,7,8]. The range of available treatments for this condition is broad and includes amputation, reconstruction with Masquelet’s induced membrane technique, large bone grafting with and without vascularization, and joint-preserving (arthroplasty) and joint-strengthening procedures (arthrodesis) [1,2,3,4,5,6,7,9,10,11,12,13,14,15,16,17,18,19,20,21,22,23]. There is a paucity of retrospective case reports and case series, and large-scale studies and robust evidence are lacking. Prospective studies are not available in the literature, and monocentric studies are not feasible due to the limited number of cases. Given the rarity and lack of good evidence, each case must be treated individually and depends largely on the available expertise of the treating professionals. For instance, in the absence of microsurgical-plastic expertise, the full spectrum of available free (arterialized) bone grafting techniques may remain inaccessible. This can result in suboptimal treatment outcomes and, ultimately, poorer hand function due to a lack of viable alternatives [12]. The objective of this study is to develop an algorithm for the treatment of large bone defects in the interval between the distal radius and carpus or proximal metacarpal. To the best of our knowledge, there is no existing study or comprehensive review of the literature and treatment algorithm that addresses the management of large bone defects in the interval between the distal radius and the metacarpus. A treatment pathway was developed based on the expertise of the authors, the case series of one of the largest hand trauma centers in Germany, and the available evidence in the literature. The optimal treatment option was determined based on currently available modern reconstruction techniques, with predictable results for hand function and quality of life (QoL). Ultimately, the orthopedic and plastic surgeon treating this rare condition will be able to work with the patient on an individualized, low but evidence-based treatment plan with somewhat predictable outcomes in terms of bone union, hand function, and QoL.

## 2. Materials and Methods

In the initial phase of this study, a retrospective analysis was conducted from January 2004 to April 2024 at our hospital. A total of eight patients with long-distance bone defects of the distal radius and/or carpus were reconstructed during this period (Table 1). A large bony defect was defined as a defect length from proximal to distal of >5 cm in the interval from the distal radius to the maximum proximal metacarpal. The length of the defect was quantified digitally based on preoperative radiographs and/or preoperative CT scans. Baseline data, including age, affected hand side, occupation, and, if available, preoperative wrist function scores, were retrospectively collected for all eight patients. The Modified Mayo Wrist Score (MMWS) and Disabilities of the Arm, Shoulder, and Hand (DASH) score were recorded in four of the eight patients. A summary of our surgical approaches in eight cases is provided in Section 3. The second step involved a comprehensive literature search, which was conducted using a range of keywords and combinations of keywords. These included “wrist reconstruction”, “fibula head”, “iliac crest”, “osseous defects”, “fibula flap”, “upper limb”, and “massive bone defects”. The identified and included studies are listed in Table 2 and were stratified according to study quality, type of reconstruction, and outcome parameters recorded, including hand and wrist function and grip strength. A descriptive statistic of our case series and the cases from the literature was performed using JASP version 0.19.0. Descriptive statistics were expressed as the mean and standard deviation.

Finally, based on our own experience and available data from the literature, we have developed an algorithm for the treatment of large bone defects in the interval between the distal radius and proximal metacarpal.

## 3. Surgical Techniques

A distinct reconstruction technique was employed in each of the eight cases that were operated upon at our institution. A brief description of each surgical technique is provided, accompanied by a list of the key elements. We subsumed our techniques to four groups: Group 1: Arthrodesis of the radius and wrist with vascularized bone flaps; Group 2: Arthrodesis of the radius and metacarpals with vascularized bone flaps; Group 3: Arthroplasty of the wrist with a vascularized fibula head; and, finally, Group 4: Partial fusion of the wrist (RFSL-Fusion) using a vascularized fibula.

### 3.1. Group 1: Arthrodesis of the Radius and Wrist with Vascularized Bone Flaps

#### Arthrodesis of the Wrist with Vascularized Fibula Flap for the Treatment of a Large Defect of the Distal Radius

**Figure 1:**
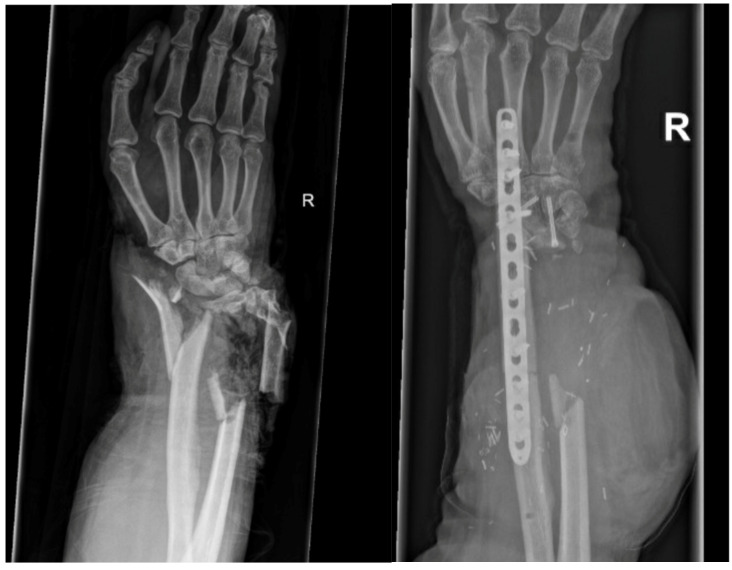
Initial X-ray after trauma (**left**) and 2 months after reconstruction with free fibula and wrist fusion (**right**).

The patient presented with a complex forearm injury sustained at work, resulting in a long-segment defect of the distal radius and ulna (Figure 1). Following radical debridement and treatment of various tendons and the radial artery, a vacuum-assisted closure (VAC) dressing was applied, and an external fixator was placed. This was followed by bony reconstruction of the 9 cm long bone defect of the distal radius using a free arterialized fibula from the contralateral lower leg and simultaneous wrist fusion due to extensive cartilage, capsule, and ligament damage to the remaining carpus. Consequently, a fusion of the scaphoid, lunate, free fibula, and the remaining radius (RFSL-Fusion) was deemed inadvisable. In October 2023, the remaining radius, free fibula, and midcarpal bone 3 were fused with a one-third tube plate. The microsurgical anastomosis of the fibula vessels to the radial artery was performed using an end-to-side technique and an end-to-end configuration to an accompanying vein. A subsequent CT scan conducted four months post-surgery demonstrated progressive bone consolidation, although this process has not yet reached completion.

### 3.2. Group 2: Arthrodesis of the Radius and Metacarpals with Vascularized Bone Flaps

#### 3.2.1. Arthrodesis of the Wrist with Vascularized Iliac Crest Bone

**Figure 2:**
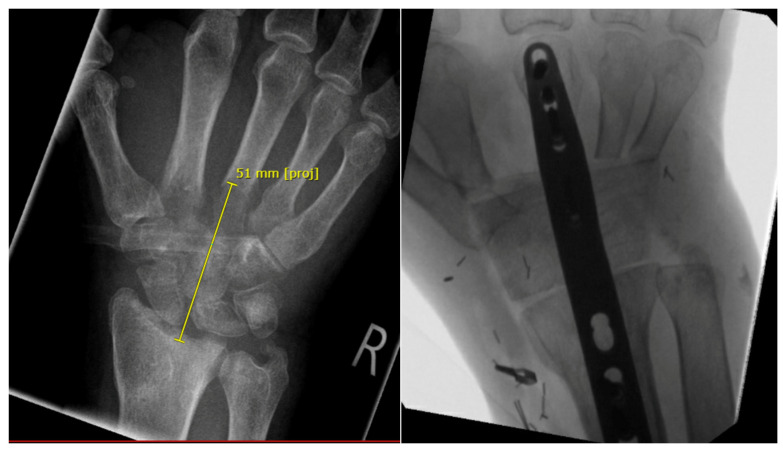
Preoperative X-ray (**left**) with osteitis of the carpal bones and the proximal metacarpal bones. Intra-operative X-ray with reconstruction of the space between the distal radius and the middle hand with a free vascularized iliac crest (**right**).

In the second case, all carpals and adjacent metacarpal bases were resected due to purulent osteomyelitis in a 64-year-old male. The resulting defect, measuring over 5 cm, was reconstructed with an arterialized iliac crest graft from the contralateral side and an LCT plate (Figure 2). A vascular anastomosis was made to the radial artery. During the healing process, it was observed that a good bony union of the radius and iliac crest graft had been achieved. In contrast, the union of the iliac crest graft and metacarpal 3 was observed to have occurred at a delayed time point. The resulting hand function was moderate to poor.

#### 3.2.2. Arthrodesis of the Distal Radius and Middle Hand Using a Triangular-Shaped Vascularized Osteocutaneous Fibula Flap

**Figure 3:**
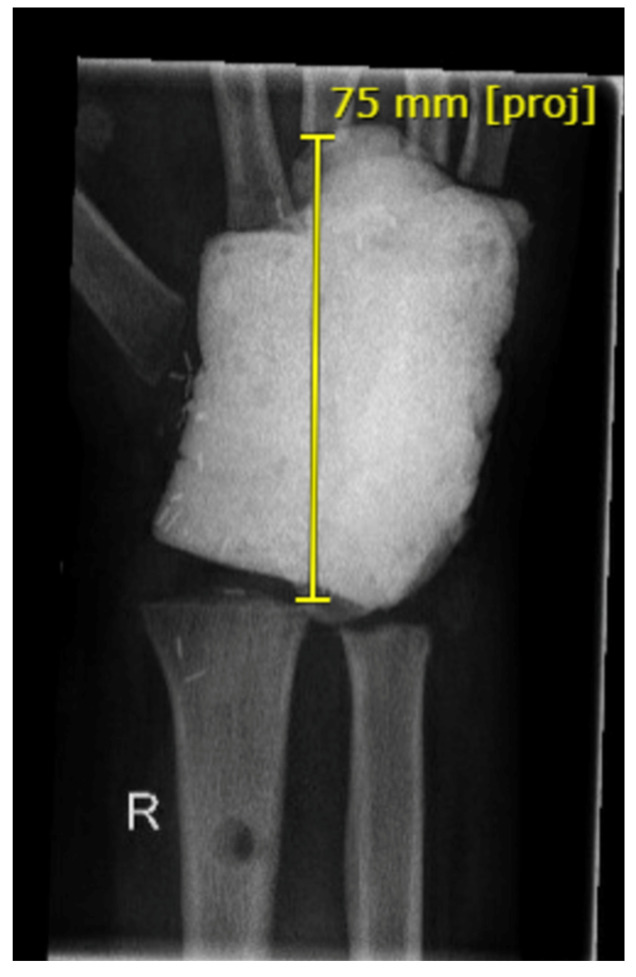
Large bone defect between distal radius and proximal metacarpal bones 2 to 5 after tumor resection. The space was filled temporarily with bone cement (Palacos).

**Figure 4:**
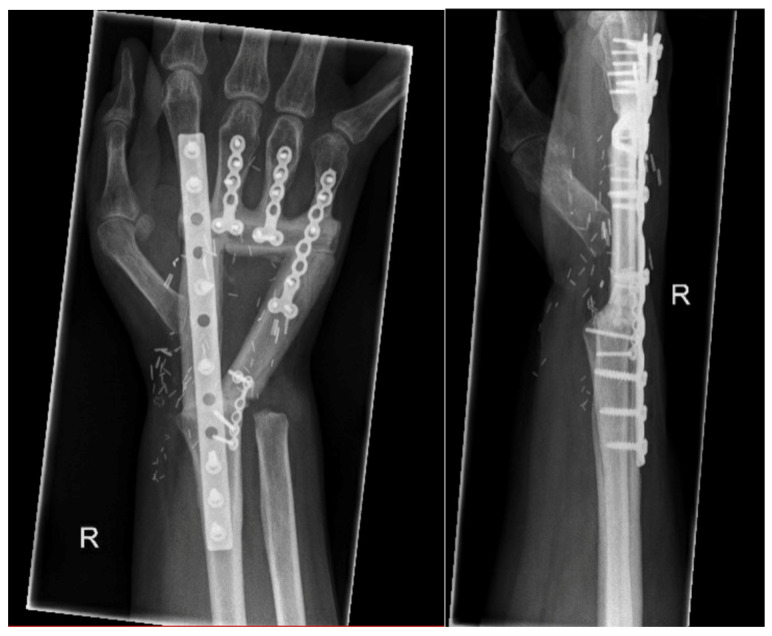
Reconstruction with a triangular shaped free osteocutaneous fibula flap and multiple fixation plates. Two years after the surgery, X-rays revealed a complete bone union.

In the third patient, a 41-year-old man, the entire wrist had to be removed due to a bone tumor, as well as the adjacent bases of the metacarpals 1–5, the distal radius, and ulna. The tumor was benign, but demonstrated aggressive growth characteristics, similar to angiomatosis. Following resection and temporary filling with bone cement, a defect of almost 8 cm remained (Figure 3). This was subsequently addressed with the transplantation of a 26 cm long osteocutaneous triangular-shaped free fibula (Figure 4). The microsurgical vascular anastomosis was established in accordance with the previously described methodology. Following a period of 1.5 years, the bone segments had fully healed.

### 3.3. Group 3: Arthroplasty of the Proximal Wrist

**Figure 5:**
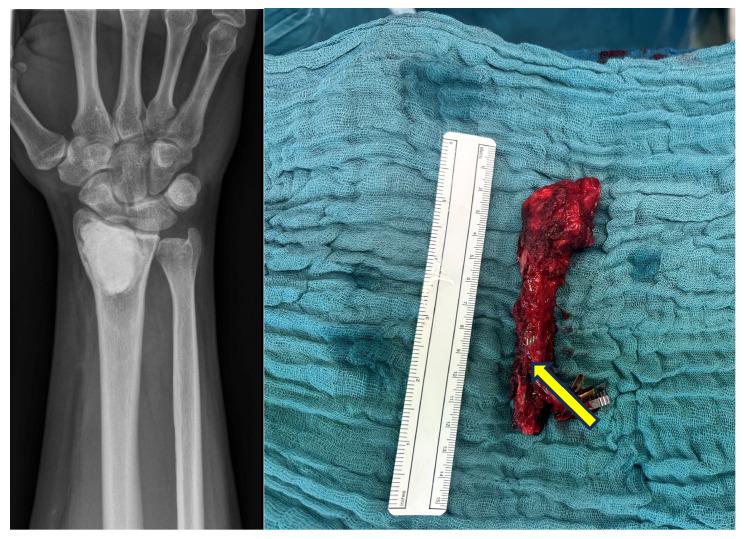
Initial findings with a cement plug in the distal radius and diagnosed second recurrence of a giant cell tumor (**left**). Elevated fibular head graft with distal vascular pedicle of the anterior tibial artery (**right**: yellow arrow).

**Figure 6:**
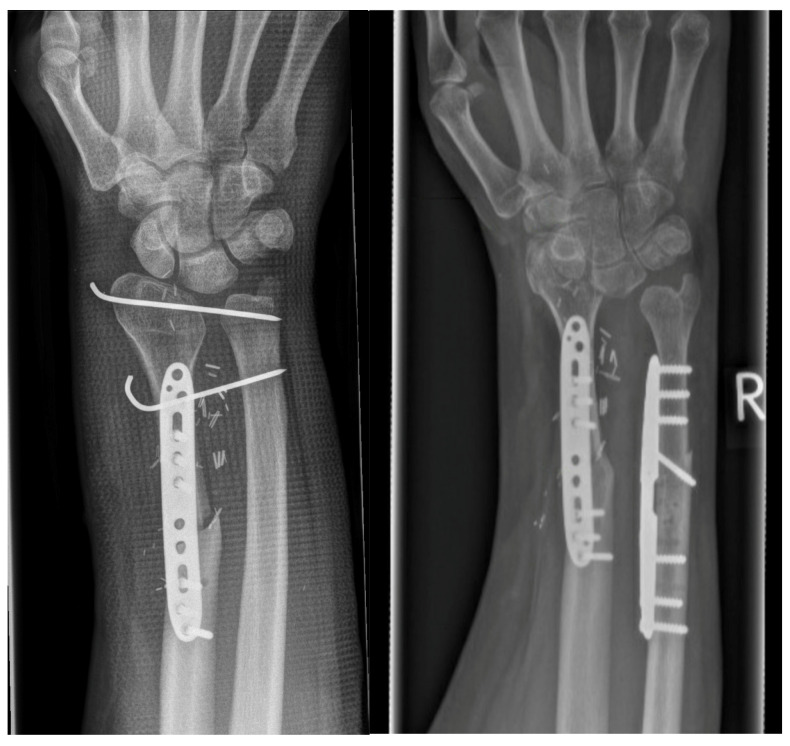
First postoperative X-ray. The fibular head graft and distal ulna were transfixed (**left**). One year later, the ulna was shortened, while wrist function remained good despite the narrowing of the new proximal wrist joint with subluxation (**right**).

A 41-year-old patient presented with a second recurrence of a giant cell tumor of the distal radius. Given the patient’s excellent wrist function, we elected not to perform a partial fusion in the form of a RFSL-fusion. Instead, the distal radius was completely resected in the healthy area and reconstructed with a 9 cm long vascularized fibula head transplant (Figure 5). The arterialization was carried out by connecting the anterior tibial artery to the radial artery in retrograde fashion. The distal radioulnar joint was transfixed, and the extrinsic ligaments from the carpus and the TFCC were reinserted to the fibula head. One year following the procedure, the fibula transplant was observed to be insufficiently long due to subluxation of the wrist. An ulnar impaction syndrome was diagnosed, necessitating a 1 cm reduction in the length of the ulna (Figure 6). The patient has since exhibited no signs of discomfort and demonstrates good wrist functionality.

### 3.4. Group 4: Fusion of Scaphoid, Lunate, Fibula and Radius (RFSL-Fusion)

**Figure 7:**
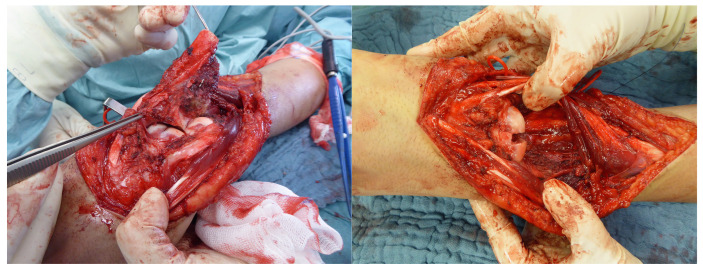
Resected distal radius with tumor (**left**). Preparation of a bony groove between the scaphoid and lunar bones for fusion (**right**).

**Figure 8:**
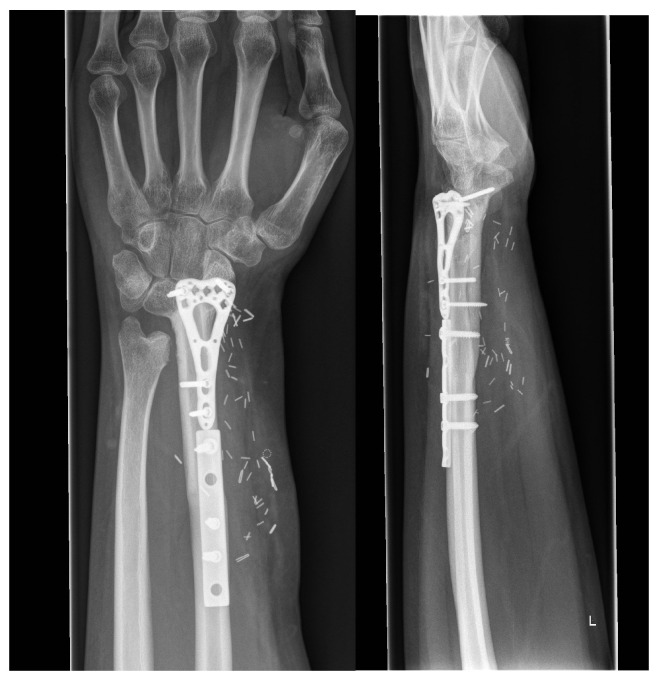
The X-ray taken 13 months later demonstrates the bony consolidation of the RFSL.

Three of the four patients underwent surgical intervention for the treatment of a giant cell tumor in the distal radius. One patient presented with an osteoblastoma in the distal radius following initial sarcoma resection (Figure 7). In all four cases, the distal radius with the articular surface was resected, and a partial fusion of the radius, scaphoid, and lunate bone with interposition of a free osteocutaneous fibula was performed. The defect length ranged between 6 and 10 cm. The distal part of the fibula graft was fused with the lunate and scaphoid (Figure 7). Additionally, the proximal fibula was fused with the remaining radius and an additional plate (Figure 8). All fusions healed, and three out of the four cases were lost to long-term follow-up, as the patients had already been treated between 2004 and 2010. The available data originate from a 2011 follow-up from our institution.

## 4. Results

### 4.1. Results from Our Own Patient Population

The follow-up period ranged from 6 to 72 months. In six of the eight patients, the large bone grafts were completely consolidated with the adjacent bone. In the patient with a free iliac crest graft (Case 2), only partial union occurred distally. In the wrist fusion case with a free fibula (Case 1), the six-month follow-up is still too short to provide a definitive assessment, but there is evidence of nearly full consolidation.

### 4.2. Results Reported in Previous Studies

A total of 19 studies (179 patients), the majority of which were case reports or case series, were identified over a period of 20 years. As the data were collected and analyzed in a highly heterogeneous manner, they have been grouped according to the techniques described by us in Table 1, Group 1–4. This involved reconstructing bony parts from the distal radius to the midcarpal joint. Only two single case–control studies were identified (Group 1 vs. Group 3; Group 3 vs. Group 4), which is indicative of the aforementioned lack of evidence. Thirteen of the nineteen papers examined the outcome after en bloc resection of the distal radius (usually due to a giant cell tumor or recurrence). This was either by arthroplasty (Group 3) with fibula head transplantation, or by partial (Group 4) or full fusion with replacement of the distal radius (Group 1). Four cases, comprising two articles, described the triangular fibula and membrane technique, in which the radius is fused over a free fibula or a free iliac crest with or without vascularization (Group 2). Osseous union was achieved in all vascularized bone grafts (90/90 cases; 100% union rate), whereas in contrast, a union was achieved in 75 of 89 cases (75/89; 84% union rate) in the non-vascularized cases. In 174 out of all reported 179 cases (97%), the primary cause of a significant bone defect was a giant cell tumor in the distal radius. Three cases were reported to have osteomyelitis of the entire wrist, one case involved an osteosarcoma in the distal radius. In one case, a failed wrist prosthesis led to a wrist arthrodesis with a free fibula flap. The range of motion of the wrists in terms of extension and flexion was found to vary considerably between different surgical procedures. In cases of wrist arthrodesis, the range of motion (extension/flexion) was found to be 0°; in contrast, in cases of wrist arthroplasty with a free fibula head, the mean range of motion (extension/flexion) was found to be 52-0-49° at the highest in 17 cases reported by Yang et al., 2017 [16].

### 4.3. Statistical Analysis of All Four Groups

A descriptive analysis was conducted on all four groups from the case series and from the literature. The range of motion (extension/flexion) was found to be the highest in group 3, followed by group 4 (Table 3). The MMWS was found to be the highest in group 3, while the DASH score was found to be the lowest in group 1. The application of interferential statistics was not feasible due to the considerable heterogeneity in group size and the absence of parameters in some patients and series.

## 5. Discussion

### 5.1. General Results

The primary etiology of extensive defects in the bony interval between the radius and metacarpal in our series and in the existing literature is neoplastic. However, additional etiologies of extensive bone defects in the hand include trauma, osteomyelitis (cases 1 and 2 in our case series), and failed wrist prosthesis. It is theoretically possible that rheumatoid arthritis and osteoarthritis may also be the cause for large osseous defects; however, no cases have been identified in the relevant literature. The graft types in our cases and in the literature were mainly vascularized and non-vascularized autologous bone grafts. Two cases were found with the non-vascularized induced membrane technique. Another technique is bone transport, which is primarily described for the lower extremity. It is also employed for the upper limb, albeit infrequently. A scoping review in 2021 demonstrated that autografts (primarily free fibulae) were superior to the induced membrane technique and bone transport in the upper limb [29]. The shortest healing time and lowest number of surgeries required to achieve final union were observed in cases where structural autografts were employed [29]. The free vascularized fibula grafting technique exhibited in a meta-analysis in 2024 a shorter time to bone union, a reduced time for external fixator application, and a lower incidence of complications compared to the other technique in lower limbs [30]. In the era of contemporary microsurgery, there is no rationale for utilizing bone transport on the upper extremity. Furthermore, a distinction was observed between vascularized and non-vascularized free fibulae by Ferreira et al. [29]. Non vascularized fibulae had higher complications compared to vascularized fibulae (13% infection and 8% fracture). But it is important to note that the non-vascularized fibulae were utilized exclusively for humeral reconstruction in their review. In general, these findings are consistent with our previous studies, which demonstrated that the healing rates of vascularized grafts are superior overall to those of non-vascularized grafts.

### 5.2. Group 1: Arthrodesis of the Radius and Wrist with Vascularized Bone Flaps

In 16 cases of distal radius bone defect reconstruction with vascularized fibula and wrist arthrodesis combined from our clinic and the literature, complete healing was demonstrated. In contrast, the series of Wang et al. with 27 free non-vascularized iliac crest grafts exhibited a non-union rate in almost a quarter of all cases. The donor-site morbidity appears to be comparable between the two techniques and is compatible with a good QoL [31,32]. Clarkson et al. compared both techniques retrospectively and found no significant differences in donor-site morbidity and DASH Scores [24]. Furthermore, all 14 free vascularized fibulae and all 13 non-vascularized free iliac crests demonstrated successful healing outcomes, in contrast to the series of studies conducted by Wang et al. The authors concluded that the non-vascularized iliac crest technique is advantageous in terms of total operating time, which is shorter than with the fibula technique. However, no significant difference could be detected [24]. Furthermore, the authors concluded that free fibular flaps should only be used for defects of more than 10 cm in length or if a skin island is required. Based on the evidence presented and our experience, we propose the use of the free vascularized fibula as a viable option. This approach offers greater versatility, with the potential to include a skin and monitor island, with minimal donor site morbidity and a lower overall incidence of non-unions. The QoL in group 1 was found to be poor with regard to the MMWS and even good in the DASH questionnaire. Nevertheless, the MMWS was only ascertained in one patient from the entire cohort (group 1; *n* = 56 patients). The Musculoskeletal Tumor Society score (MSTS) of 54 patients in the literature (group 1) was 96% and 90%, respectively. However, the assessment via MSTS does not take into account the patient’s quality of life or mental status, but rather the function of the affected limb [33]. Considering all of the data, a good quality of life, short healing time, and low non-union rates can be achieved in the reconstruction of long distal radius defects with complete fusion of the wrist to the distal radius using vascularized autologous bone grafts. In the event that a completely suspended extension/flexion is not feasible, alternative options should be discussed with the patient, provided that the midcarpal joint surfaces are intact.

### 5.3. Group 2: Arthrodesis of the Radius and Metacarpals with Vascularized Bone Flaps

The challenge presented by a large bone defect between the proximal metacarpal and distal radius is to establish an anatomically correct connection between the narrow end of the radius and all four metacarpals. Previous surgical approaches described the use of a double fibula, whereby two metacarpals are positioned in a Y-shaped configuration on one fibular column each. This can easily lead to axial deviations and rotational errors of the fingers [7]. In autumn 2020, the first operation utilizing a novel design, the triangular-shaped free osteocutaneous fibula (Figure 4), was conducted at our center [4]. In 2022, a working group from Australia published a technical note describing a similar concept in two cases [24]. The combined three cases demonstrated excellent healing outcomes and satisfactory hand function. While the free iliac crest can be considered as an alternative, it did not prove effective in our own application, where incomplete bone healing at the distal end was observed with only moderate grip strength (50%). The vascularized induced membrane technique, as described by Inui et al. in two cases, can also be applied [20]. However, this technique involves several additional surgical steps, longer hospitalization, and the union rate (50%) was not as high as in the vascularized triangular fibula group (100%). The quality of life (QoL) could only be determined for one patient, thus precluding any generalizable assertion regarding Group 2 or in comparison with the other groups. Large osseous defects in the interval between radius and metacarpals are extremely rare. In regard to bone healing and the anatomically correct reconstruction of the remaining metacarpal bones, the triangular technique of the free fibula appears to be the most effective approach, based on our analysis. However, the data situation is insufficient to draw any conclusions regarding the quality of life.

### 5.4. Group 3: Arthroplasty of the Proximal Wrist

The largest body of evidence in the literature concerns Group 3 of Table 1 and Table 2, which involves the reconstruction of the distal radius and radiocarpal joint with a free fibula head (arthroplasty), both vascularized and non-vascularized (*n* = 83). This technique requires the wrist bones to be intact and only the distal radius portion, including the joint surface, needs to be replaced. With this technique, postoperative ranges of motion for extension and flexion of >60° can be achieved with a relatively high degree of certainty and good QoL [2,5,6,10,12,16,18,19]. These findings are analogous to those observed in the postoperative range of motion for wrist extension and flexion, which demonstrated a mean value of 88.56° ± 11.71° and the highest MMWS (72.59 ± 5.40) among all groups. In contrast, the DASH score for this group was higher, indicating a comparatively unfavorable outcome. This discrepancy may be attributed to the fact that the MMWS is primarily determined by grip strength, rather than pain, in contrast to the DASH score [34]. It is essential to acknowledge that this technique is theoretically associated with an increased risk of donor morbidity. This may manifest as at least a transient peroneal paresis with foot drop. The cases described in Group 3, Table 2, in the literature reported three peroneal lesions, and one that recovered in the final follow-up. Other reported complications are subluxation of the wrist or of the distal radioulnar joint, and narrowing of the wrist space. No cases exhibited evidence of knee instability. In the event of a persistent peroneal lesion, surgical intervention may be indicated. This may entail a stirrup plasty or, as a last resort, the stiffening of the ankle joint. Subluxations are relatively common due to the incomplete replacement of the joint surface of the fibula head. Therefore, a temporary fixation of the carpal bone to the head of the fibula is recommended, as described by Innocenti et al. [35]. As we advocate the use of vascularized autografts for several reasons, there seems to be no significant difference between non vascularized fibula heads and vascularized fibula head transplants regarding bone unions. Only Qu et al. reported, in 2018, a malunion in a non-vascularized arthroplasty with a fibula head [18]. It is plausible that the reduced number of osteotomy sites necessitated by this procedure, in comparison to a bony interposition, is a contributing factor. Furthermore, it is frequently asserted by authors that bone defects exceeding 6 cm necessitate the use of vascularized bone autografts. However, there is a paucity of compelling scientific evidence to support these recommendations, as evidenced by a systematic review published in 2017 [36]. In contrast, there is evidence that vascularized bone grafts can result in superior functional and pain scores, as well as a significantly earlier bony union compared to non-vascularized bone grafts [36]. A review of the literature indicates that wrist arthroplasty may be less effective than wrist arthrodesis in terms of grip strength and possible long-term complications such as subluxation of the wrist and radiocarpal arthrosis. But in light of the aforementioned considerations, wrist arthroplasty with a vascularized fibular head autograft may be a suitable option, offering superior wrist function regarding extension and flexion, satisfactory grip strength, and a relatively low complication rate (Table 3). Furthermore, wrist arthrodesis remains a viable alternative in the event of failure of the fibular head autograft reconstruction [5,6]. However, the technique of fibular head raising is highly demanding, and the patient should be fully informed and offered possible alternatives such as a RFSL fusion.

### 5.5. Group 4: Fusion of the Proximal Row of the Wrist with the Distal Radius (RFSL Fusion)

The indication for fusion of the radius with the proximal carpal row bones, scaphoid and lunate, is based on the literature in the case of a destroyed radial joint surface [3,26,27]. The procedure has the advantage that, with careful dissection, no (mid)carpal dislocation is expected [3,26]. In contrast, the obvious disadvantage is the limitation of wrist mobility compared to wrist arthroplasty (Table 3) [27]. Zhu et al. demonstrated, in a retrospective case–control study, that arthroplasty (*n* = 7) regarding wrist function (71.6°  ±  16.1° ROM Extension/Flexion) yielded superior outcomes compared to partial wrist fusion (*n* = 7; 55.9°  ±  7.5°), with a significant improvement observed at the 3.9-year follow-up (t  =  2.34, P  =  0.037) [27]. These findings are analogous to those previously reported, and vice versa. The donor site morbidity was overall lower due to the fact that the common peroneal nerve is left untouched in group 4. Additionally, the MMWS was slightly lower in group 4 compared to group 3. The mean range of motion regarding extension and flexion was >30° less than that observed in Group 3 (Group 4: 53.00° ± 9.04° vs. Group 3: 88.56° ± 11.71°). It is our contention that partial fusion of the carpal bones with a fibula flap should preferably be performed in selected cases in order to preserve functionality as much as possible. Consequently, the procedure of carpal joint reconstruction using a free fibula head should be discussed with the patient first. Even in the case of a poor clinical and radiological outcome of arthroplasty, a change of approach to partial fusion can be performed without any problems. Nevertheless, the higher probability of a re-operation and possible complications, such as foot drop and subluxations, must be honestly discussed with the patient.

### 5.6. Limitations of the Study

This study has several limitations. As previously stated, our monocentric case series does not permit a definitive statistical analysis. Furthermore, the statistical analysis of all cases should be interpreted with caution due to the heterogeneity of cases per group and the lack of collected parameters. As a result, an interferential statistical analysis was not feasible, which limits the validity of the result accordingly. In regard to the free fibula head, it is evident that a selection bias was present on behalf of the first author. Surgery, performed initially at this institution, yielded a relatively favorable outcome, with the exception of chronic subluxation accompanied by ulnar impaction syndrome. Had the procedure resulted in a foot drop, our assessment might have differed. However, the existing literature demonstrates comparable favorable outcomes. The paucity of cases and the tenuousness of the evidence in this study, when considered alongside the evidence available in the extant literature, give rise to a concomitant limitation of the study’s validity.

## 6. Conclusions

**Figure 9:**
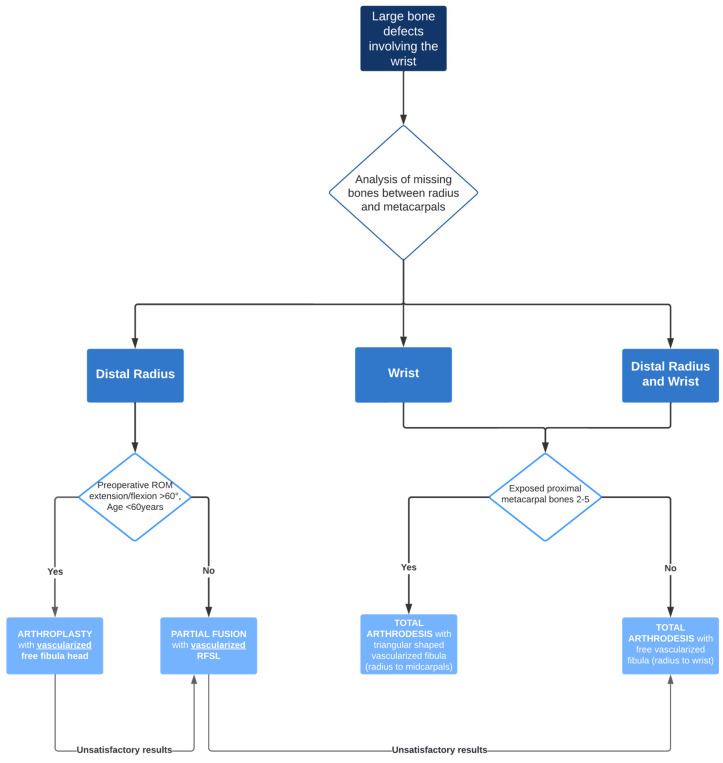
Treatment algorithm for free vascularized bone reconstruction in rare large osseous defects involving the wrist.

A surgical treatment algorithm (Figure 9) for the management of large bone defects in the wrist is proposed, based on the findings of our study and those reported in the literature.

## Figures and Tables

**Table 1:** Results after large reconstruction of bony defects in the wrist.

		Localization of the Defect	Pathogenesis	Follow-Up	Age	Defect Lentgh	Graft Type	Reconstruction Concept	Pre-operative ROM Extension/Flexion Pronation/Supination	Post-Operative ROM Extension/Flexion Pronation/Supination	Grip Strength	Quality of Life	Bone Consolidation
***GROUP 1***	Case 1	*Distal radius, parts of the proximal wrist*	*Trauma*	*6 month*	*32*	*9 cm*	*Vascularized fibula*	*Wrist arthrodesis*	*N/A* *N/A*	*0-0-0°* *40-0-50°*	*60%*	*MMWS = 50* *DASH = 24*	*yes*
***GROUP 2***	Case 2	*Complete wrist, distal radius and proximal metacarpals*	*Osteomyelitis*	*47 month*	*64*	*>5 cm*	*Vascularized iliac crest*	*Wrist arthrodesis*	*N/A* *N/A*	*0-0-0°* *50-0-35°*	*50%*	*(N/A)*	*partial*
	Case 3	*Complete wrist, distal radius and proximal metacarpals*	*Benign, aggressive bone tumor*	*53*	*41*	*8 cm*	*Vascularized osteocutaneous triangular fibula*	*Wrist arthrodesis*	*N/A* *N/A*	*0-0-0°* *80-0-40°*	*70%*	*MMWS = 45* *DASH = 30*	*yes*
***GROUP 3***	Case 4	*Distal radius involving the radiocarpal surface*	*Giant cell tumor*	*14*	*41*	*8 cm*	*Vascularized fibula*	*Wrist arthroplasty*	*60-0-50°* *N/A*	*30-0-40°* *80-0-70°*	*65%*	*MMWS = 70* *DASH = 17*	*yes*
***GROUP 4***	Case 5	*Distal radius involving the radiocarpal surface*	*Sarcoma, secondary osteoblastoma*	*61*	*20*	*9*	*Vascularized fibula*	*RFSL-Fusion*	*N/A* *N/A*	*25-0-30* *50-0-60*	*70%*	*MMWS = 65* *DASH = 24*	*yes*
	Case 6	*Distal radius involving the radiocarpal surface*	*Giant cell tumor*	*72*	*45*	*6*	*Vascularized fibula*	*RFSL-Fusion*	*60-0-60* *70-0-70*	*30-0-30* *70-0-50*	*80%*	*(N/A)*	*yes*
	Case 7	*Distal radius involving the radiocarpal surface*	*Giant cell tumor*	*25*	*38*	*8*	*Vascularized fibula*	*RFSL-Fusion*	*50-0-50* *90-0-50*	*40-0-0* *80-0-70*	*80%*	*(N/A)*	*yes*
	Case 8	*Distal radius involving the radiocarpal surface*	*Giant cell tumor*	*6*	*40*	*7.5*	*Vascularized fibula*	*RFSL-Fusion*	*70-0-70* *80-0-80*	*30-0-10* *50-0-50*	*60%*	*(N/A)*	*yes*

**Table 2:** Surgical approaches by defect interval and results reported in previous studies.

		Study Type	Patients/Cases	Pathogenesis	Localization of the Defect	Graft Type	Reconstruction Concept	Consolidation	Pre-Operative ROM Extension/Flexion Pronation/Supination	Post-Operative ROM Extension/Flexion Pronation/Supination	Grip Strength	Quality of Life	Level of Evidence
***GROUP 1 (n = 55)***	Ebinger et al., 2003 [8]	*Case report*	*1*	*Failed wrist prothesis*	*Distal radius*	*Free vascularized fibula*	*Arthrodesis*	*1/1*	*N/A* *N/A*	*0-0-0°* *70-0-75°*	*85%*	*N/A*	*4*
	Wang et al., 2017 [23]	*Cohort study*	*27*	*Giant cell tumor*	*Distal radius*	***Non** vascularized iliac crest*	*Arthrodesis*	21/27	*N/A* *N/A*	*N/A* Mean 113°	51%	*Musculoskeletal Tumour Society functional classification (MSTS) = 96%* *DASH = 9*	4
	Clarkson et al., 2013 [24]	*Case–control study*	*27*	*Giant cell tumor*	*Distal radius*	*Free vascularized fibula **vs. Non** vascularized iliac crest*	*Arthrodesis*	27/27	*N/A* *N/A*	*N/A* *N/A*	*N/A*	*MSTS = 90%* *DASH = 17*	3
***GROUP 2 (n = 4)***	Graham et al., 2022 [7]	*Case report, Technical note*	*2*	*Osteomyelitis, Giant cell tumor*	*Distal radius to proximal middle hand*	*Vascularized triangular shaped fibula*	*Arthrodesis*	*2/2*	*N/A* *N/A*	*N/A* *N/A*	*N/A*	*N/A*	*4*
	Inui et al., 2020 [20]	*Case report*	*2*	*Osteomyelitis*	*Carpus*	***Non** vascularized induced membrane technique*	*Arthrodesis*	*1/2*	*N/A* *N/A*	*N/A* *N/A*	*80%*	*N/A*	*4*
***GROUP 3 (n = 82)***	Mays et al., 2010 [10]	*Case report*	*1*	*Giant cell tumor*	*Distal radius and proximal row*	*Free vascularized fibula head*	*Arthroplasty*	*1/1*	*N/A* *N/A*	*35-0-45°* *N/A*	*80%*	*N/A*	*4*
	Saini et al., 2011 [25]	*Cohort study*	*12*	*Giant cell tumor*	*Distal radius*	*Non vascularized fibula head*	*Arthroplasty*	*9/12*	*N/A* *N/A*	*31-0-42°* *37-0-52°*	* 71% (42–86%) *	*MSTS = 91%*	*4*
	Chung et al., 2013 [12]	*Cohort study*	*12*	*Giant cell tumor*	*Distal radius*	*Free vascularized fibula head*	*Arthroplasty*	*12/12*	*N/A* *N/A*	*Mean 73°* *Mean 102.9°*	*57.3%*	*N/A*	*4*
	Scaglioni et al., 2014 [2]	*Case series*	*3*	*Giant cell tumor, osteosarcoma*	*Distal radius*	*Free vascularized fibula head*	*Arthroplasty*	*3/3*	*N/A* *N/A*	*32-0-72°* *83-0-38°*	*N/A*	*N/A*	*4*
	Yang et al., 2016 [16]	*Cohort study*	*17*	*Giant cell tumor*	*Distal radius*	*Free vascularized fibula head*	*Arthroplasty*	17/17	*N/A* *N/A*	52-0-49° *N/A*	77.2°	*MMWS = 77.3*	4
	Liu et al., 2019 [6]	*Cohort study*	*26*	*Giant cell tumor*	*Distal radius*	*Free vascularized fibula head*	*Arthroplasty*	26/26	*44-0-52°* *42-0-45°*	32-0-42° 22-0-31°	71%	*DASH = 33.3*	4
	Barik et al., 2020 [19]	*Cohort study*	*11*	*Giant cell tumor*	*Distal radius*	***Non** vascularized fibula head*	*Arthroplasty*	11/11	*N/A* *N/A*	47-0-37° 64-0-57°	51%	*MMWS = 66.4*	4
***GROUP 4 (n = 3)***	Franz et al., 2010 [1]	*Case report*	*1*	*Giant cell tumor*	*Distal radius*	*Free vascularized fibula*	*RFSL-Fusion*	*1/1*	*30-0-40°* *N/A*	*40-0-20°* *70-0-80°*	*75%*	*N/A*	*4*
	Bickert et al., 2002 [26]	*Case series*	*2*	*Giant cell tumor*		*Free vascularized fibula*	*RFSL-Fusion*	*2/2*	*N/A* *N/A*	*22.5-0-35°* *62.5-0-70°*	*64.5%*	*N/A*	
***GROUP 1 vs. 3 (n = 21)***	Qu et al., 2019 [18]	*Case–control study*	*21*	*Giant cell tumor*	*Distal radius*	***Non** vascularized fibula head*	*Arthroplasty (n = 13)* *vs.* *Total wrist arthrodesis (n = 8)*	20/21	*N/A* *N/A*	*N/A* *N/A*	40% (*n* = 13) vs. 71% (*n* = 8)	*MSTS = 93%* *vs.* *83%*	3
***GROUP 3 vs. 4 (n = 14)***	Zhu et al., 2013 [27]	*Case–control study*	*14*	*Giant cell tumor*	*Distal radius*	*Vascularized and non-vascularized proximal fibula*	*Arthroplasty (n = 7) vs. partial wrist arthrodesis (n = 7)*	14/14	*N/A* *N/A*	* 71.6 ± 16.1° vs. * * 55.9 ± 7.5° * * 140 ± 14.7° vs. * * 127.6 ± 14.2° *	* 59.2 * *± 13.7%* * vs. * *76.5 ± 4.6%*	*MSTS =* * 25.9 ± 1.46 * *vs.* *25.6 ± 0.78*	3
***NO GROUP***	Wood et al., 2006 [28]	*Expert opinion*	*N/A*	*various*	*various*	*various*	*various*	*N/A*	*N/A* *N/A*	*N/A* *N/A*	*N/A*	*N/A*	*5*
	Malizos et al., 2010 [9]	*Review, expert opinion*	*N/A*	*various*	*various*	*various*	*various*	*N/A*	*N/A* *N/A*	*N/A* *N/A*	*N/A*	*N/A*	*5*
	Liu et al., 2021 [5]	*Review*	*N/A*	*various*	*various*	*various*	*various*	*N/A*	*N/A* *N/A*	*N/A* *N/A*	*N/A*	*N/A*	*4*

**Table 3:** Descriptive analysis of all cases.

Group	Frequency	Valid Percent	ROM (Mean) Extension/Flexion	ROM (Mean) Pronation/Supination	DASH (Mean)	MMWS (Mean)
1	56	36.842%	0°	113.31° ± 7.44°	13.20 ± 4.26	55 ± N/A
2	6	3.947%	0°	102.50° ± 24.75°	30.00 ± N/A	45 ± N/A
3	83	54.605%	88.56° ± 11.71°	85.02° ± 28.79°	32.41 ± 3.08	72.59 ± 5.40
4	7	4.605%	53.00° ± 9.04°	128.00° ± 19.11°	24.00 ± N/A	65 ± N/A

## Data Availability

The datasets used and/or analyzed during the current study are available from the corresponding author on reasonable request.
